# Engaging Stem Cells for Customized Tendon Regeneration

**DOI:** 10.1155/2012/309187

**Published:** 2012-05-16

**Authors:** Hatim Thaker, Arun K. Sharma

**Affiliations:** ^1^Division of Pediatric Urology, Children's Memorial Hospital of Chicago, Chicago, IL, USA; ^2^Department of Urology, Northwestern University Feinberg School of Medicine, Chicago, IL 60611, USA; ^3^Institute for BioNanotechnology in Medicine (IBNAM), 303 East Superior Street, Northwestern University, IBNAM 11-113, Chicago, IL 60611, USA

## Abstract

The need for a consistent therapeutic approach to tendon injury repair is long overdue. Patients with tendon microtears or full ruptures are eligible for a wide range of invasive and non invasive interventions, often subjectively decided by the physician. Surgery produces the best outcomes, and while studies have been conducted to optimize graft constructs and to track outcomes, the data from these studies have been inconclusive on the whole. What has been established is a clear understanding of healthy tendon architecture and the inherent process of healing. With this knowledge, tissue regeneration efforts have achieved immense progress in scaffold design, cell line selection, and, more recently, the appropriate use of cytokines and growth factors. This paper evaluates the plasticity of bone-marrow-derived stem cells and the elasticity of recently developed biomaterials towards tendon regeneration efforts. Mesenchymal stem cells (MSCs), hematopoietic progenitor cells, and poly(1,8-octanediol co-citrate) scaffolds (POC) are discussed in the context of established grafting strategies. With POC scaffolds to cradle the growth of MSCs and hematopoietic progenitor cells, developing a fibroelastic network guided by cytokines and growth factors may contribute towards consistent graft constructs, enhanced functionality, and better patient outcomes.

## 1. Introduction

Sports-related tendon and ligament injuries account for a significant portion of patient presentations, accounting for many physician hours in primary care, radiology, orthopedics, and physical therapy. The function of concentrating muscle force renders tendons and ligaments susceptible to overuse syndromes and stress injuries, with pathologies spanning across three grades [1]. These are overstretching (grade I, no pain and,no joint instability), partial tears (grade II, severe pain with joint instability), and complete tears (grade III, severe pain during injury, followed by no pain). Complete tears occur most often within the substance of collagen fibers, particularly during episodes of fast loading onto tendons and ligaments. In addition, degeneration and rupture of tendons have been associated with hypovascularity in certain regions of tissues like the posterior tibial tendon [2]. Osseous insertion points are hypervascular, in contrast to other regions prone to stress and pressure, which are avascular. This explains the tendency of tendons to rupture within the substance 

### 1.1. Inflammatory Cells and Cytokines Drive Tendon Healing

Upon tissue damage, blood vessels rupture and the exposed endothelium trigger the coagulation cascade at the site of injury, producing a hematoma. The hematoma serves to concentrate fibrin and platelets, with the latter releasing platelet-derived growth factor (PDGF), transforming growth factor beta (TGF-*β*), insulin-like growth factor-1,-2 (IGF-1,-2), and various cytokines to initiate localized inflammation and establish a chemotatic gradient. In response to the chemoattractants, neutrophils undergo diapedesis, augment the levels of TGF-*β*, and concurrently release additional factors such as basic fibroblast growth factor (bFGF) and vascular endothelial growth factor (VEGF). Meanwhile, histamine and bradykinin from platelets and neutrophils promote the formation of prostaglandins (PGE1 and PGE2), which collectively present as symptoms of pain and tenderness [3]. After the acute inflammatory phase, the level of concentrated growth factors allows the transition into the reparative stage, occurring through either an extrinsic or intrinsic pathway. 

In the extrinsic tendon repair scheme, the tear repairs itself through granulation tissue formation [4]. A scar develops after initial macrophage phagocytosis of debris, followed by activation of dormant fibroblasts, fibroblast migration and collagen deposition at day three after injury. Lactate from tissue hypoxia is the impetus for collagen deposition [5]. Macrophages respond to high lactate levels and stimulate fibroblasts to lay down extracellular matrix components, either directly or through TGF-*β*. By day seven, angiogenesis driven by VEGF is noticeable in order to support fibroblast activity [6]. Unfortunately, collagen fibers deposited under the extrinsic repair pathway lack organization [7] due to abnormal cross-linking and a predominance of type III collagen. The high level of retained glycosaminoglycans results in a weaker, fibrotic tendon that is less able to glide smoothly within its sheath [8]. 

In contrast, the intrinsic repair mechanism mimics embryonic tendon formation [9]. In this system, fibroblasts migrate to the site of defect and are responsible for synthesizing collagen fibrils of a single variety. Procollagen from the rough endoplasmic reticulum is cleaved into tropocollagen, and, instead of linking to form collagen fibers directly, they are added to collagen fibril segments first. These fibril segments are larger than tropocollagen molecules, which upon incorporation into damaged collagen at the ruptured ends, maintain the original and intended orientation of subunits, as seen in organized embryonic fibrillogenesis [10–13]. The final stages of repair start 6–8 weeks after injury and last for months thereafter. Type III collagen is replaced by type I collagen, and a reduction in cellularity and water content allows the healing tendon to approach the morphology of normal functioning tissue.

 Nutrition to the tendon is critically important in both normal physiology and healing after pathology [14]. Typically, intrasynovial tendon are supported by intrinsic cells found in the single-cell-layered synovial membrane. This membrane exists over the tendon (endotenon), on the parietal surface of the tendon sheath (epitenon), and within the adventitia (paratenon). Extrasynovial tendons receive nourishment from growth factors and cytokines available in the paratenon and systemic vasculature. These factors include IGF-1, PDGF, bFGF, each especially vital in the early and immediate stages of healing. They initiate and guide fibroblast activation, proliferation, and migration. TGF-*β* and VEGF display their importance in the remodeling phase, prompting angiogenesis [3].

## 2. Current Therapeutic Strategies

The approach to treatment in acute soft tissue trauma relies heavily on the patient's history, signs, and symptoms including the grade of injury, and their goals of usage after therapy. Initially, clinical evaluation determines the grade of injury and the level of instability to the joint. Radiologic techniques of ultrasound and magnetic resonance imaging assist the diagnosis, after which the patient is considered for conservative or surgical treatment. For all injuries, initial management is aimed at controlling edema, increasing stability and decreasing pain and inflammation. These goals can be achieved by protection, rest, ice, compression, elevation and support. 

### 2.1. Non Surgical Approach

For small tears or overuse injuries, physicians opt for conservative therapy to strengthen and stretch the tendon. After all, older patients with co morbidities and arthritis are less eligible for surgery, particularly if the therapeutic intent is achieving stability over high-intensity, sport-related joint use [15]. Some injuries are even considered irreparable [16]. Non surgical rehabilitation involves immobilization and strengthening of the muscles around the joint. This approach relies on the intrinsic and extrinsic mechanisms of repair as discussed above. While movement, stretching or heat is not recommended during the inflammatory phase (weeks 0–3), a gradual progression towards controlled weight-bearing exercise and plyometrics is allowed for the reparative and remodeling stages (weeks 3–12). At a cellular level, stretching and strengthening encourage collagen synthesis [17]. Without appropriate physical therapy, collagen fibrils are not arranged linearly and yield a weak scar prone to further injury. Early mobilization of the joint followed by late passive or active motility prevents complications like adhesions to the synovium [18].

### 2.2. Surgical Approach

For young patients hoping to achieve pre injury conditions of use, surgical interventions can reconstitute function up to 98%. Surgery, however, is not without difficulties. Suture techniques are numerous, and selecting an appropriate graft is a further challenge. For anterior cruciate ligament injuries, gracilis hamstring autografts are commonly harvested, but great attention must be given to attain appropriate tension and fixation of the graft in surgery. A flaccid graft, or one over-tightened, would compromise stability and range of motion. Still, surgery remains an optimal choice. In Achilles tendon ruptures, surgical treatment was associated with a lower risk of re rupture compared to other interventions [19]. In addition, complications such as infection, nerve damage, adhesions, and disturbed skin sensibility must be considered with open surgery [20–23]. Recent insights have revealed that percutaneous approaches to tendon repair tend to minimize infection and improve patient satisfaction despite the inability of the surgeon to visualize the defect [24]. While the cosmetic difference between the open and percutaneous method is noteworthy, there is still contention as to whether the percutaneous approach has an effect on the rates of tendon re rupture, the residual gap between blunted ends, or the level of nerve injury [25–27]. 

Recently, therapies using autologous platelet-rich plasma (PRP) injections are being explored. PRP mimics the high concentration of platelets in the hematoma surrounding the site of injury [28]. A bolus of PRP includes platelets (up to six times more concentrated than in blood), TGF-*β*, PDGF, VEGF, IGF-1, fibrin, fibronectin, vitronectin, thrombospondin, osteonectin, and osteocalcin [29]. While this appears mechanistically ideal, randomized controlled trials have shown little to no improvement on disease progress [30, 31]. The appropriate dosage of PRP for soft tissue injuries remains unknown, and the effects rely heavily on anatomical site and grade of injury as well [29]. 

For certain patient populations, especially those with complete ruptures, surgery is the standard of care for tendon and ligament injuries [32]. Even with advances in surgery, there still remains an inherent need to establish physiological function with a high degree of reproducible outcomes for patients. While the lack of clear post operative rehabilitation protocols may contribute to this [33], findings from a meta-analysis by Mohtadi et al. [34] elaborate on the issue. In comparing the outcomes of anterior cruciate ligament repairs with autologous hamstring or patellar tendons, no differences were found in function or rates of rerupture. However, patellar grafts were shown to produce a more stable knee, at the expense of anterior knee discomfort and decreased range of extension. Patients with a hamstring graft had weaker knees and a decreased range of flexion range and strength. Evidently, neither graft is optimal. Looking forward, the open surgical strategy invites the use of biomaterials and autologous stem cells to enhance native repair mechanisms, a reconstruction strategy that may address this uncertainty in patient outcomes.

## 3. Mesenchymal Stem Cells as a Candidate for Tendon and Ligament Repair

Tenocytes, or elongated fibroblast cells resident to the tendon, are responsible for the generation of collagen fibers in fibrillogenesis but remain fairly dormant in normal usage conditions. Upon injury, they are activated by the inflammatory response for collagen deposition. To conduct this function, tenocytes are assisted by tendon-derived stem cells (TDSCs). Studies have shown that TDSCs induce tenocyte differentiation upon mechanical stimulation [35].

From the regenerative standpoint, ligaments and tendons can be considered structurally similar. Type I collagen predominates in both fibroelastic structures, with the remaining substance consisting of fibroblasts, ground substance, elastin, and water [36]. Ligaments have a slightly reduced collagen fibril percentage, but a higher elastin and proteoglycan component compared to tendons. Moreover, within the category of tendons, no significant differences have been documented in construct or elasticity between males and females [37]. Nonetheless, their major difference is in function, not composition. By healing the collagen fibers similarly in tendons and ligaments, construction of grafts becomes more efficient to reinstate sustained tensile loads after injury. 

The regenerative capacity of mesenchymal stem cells is now well established in a multitude of fields, including orthopedics [38–42]. Although rare in the bone marrow, occurring at a rate of one in 100,000 nucleated cells [43], MSCs possess a high, though not indefinite, proliferation capacity that compensates for their rarity. They are capable of dividing 24–40 times to expand the cell population to well above one million cells [44]. MSCs express cell surface markers CD29, CD44, CD105 and CD166 and are negative for hematopoietic markers such as CD14, CD34, and CD40. MSCs are also negative for leukocyte common antigen CD45, suggesting that these stem cells cannot stimulate allogeneic lymphocyte proliferation and will thus avoid immune rejection [45]. Their distinction from hematopoietic cells of the bone marrow allows ease of isolation through flow cytometry, making MSCs readily available for use [46].

One great advantage of MSCs, as described by Pittenger et al. [47], is that they do not differentiate spontaneously during *in vitro* culture. This permits a controlled micro environment, such as the target tissue itself, to dictate the differentiation of MSCs after implantation. From a regeneration perspective, MSCs display an immunomodulatory effect [48] that includes the secretion of cytokines to initiate tissue regeneration [49]. At present, multipotent MSCs have been differentiated into neural [50], cardiac, osteogenic, and adipogenic lineages. The plasticity inherent in MSCs makes this cell a prime candidate for soft tissue regeneration, particularly since they support orthopedic healing with minimal complications [51]. 

### 3.1. MSCs Appear Similar to TDSCs

In a characterization study, undifferentiated TDSCs were compared to bone-marrow-derived mesenchymal stem cells (BM-MSCs) as also important in regeneration. TDSCs were found to have a higher clonogenicity and proliferation rate, and expression profiles revealed a higher level of tenomodulin, scleraxis, COL1A1, alkaline phosphatase, COL2A1, and biglycan mRNA expression compared to BM-MSCs [52]. While TDSCs appear more suited for regeneration of soft tissue, these soft tissue-specific mRNAs were not absent from MSCs, indicating their capacity to act like TDSCs. A summary comparing TDSCs and MSCs presented in Table 1.

Given the capacity of MSCs to be influenced by their microenvironment, it is expected that MSCs in the context of the tendon sheath would up regulate relevant protein content to that found in TDSCs. Direct and compelling evidence has already shown the ability of MSCs to differentiate into tenocytes [53, 54]. In addition, MSCs are more easily isolated and banked from bone marrow compared to TDSCs, which are harvested from fragile peritendinous connective tissue [55]. From a therapeutic perspective, isolating connective tissue from the site of injury requires two invasive procedures at the same site, whereas bone marrow aspiration will not aggravate the healing process at the tendon rupture location. 

In an effort to reconstitute flexor tendon tissue, Kryger et al. [55] demonstrated the similarities between differentiated epitenon tenocytes and BM-MSCs. Morphologically, BM-MSCs and tenocytes were both spindle shaped, and both stained strongly for collagen 1 and 3 [56]. Like BM-MSCs, tenocytes showed no senescence across multiple passage rounds, illustrating the long-term capacity of both cell types to support regeneration in grafts. If growth was not sustained over the course of healing, applications of MSCs would be stunted from arrested growth in the more complex synovial environment. When seeded upon acellular tendon grafts, both cell types retained their collagen architecture *in vivo* with an inflammatory response equal to that of controls. These characteristics support the use of BM-MSCs for tendon regeneration, particularly when part of seeded scaffolds [57–59]. Like TDSCs, MSCs have been induced to differentiate to tenocytes through the Wnt signaling pathway and cyclic mechanical stimulation that mimics normal processes [60]. Interestingly, platelet-rich plasma (PRP) was found to stimulate both MSCs and TDSCs. PRP enhanced MSC proliferation towards soft tissue lineages and induced TDSC differentiation into tenocytes [61, 62]. True validation that MSCs are a viable candidate would illustrate a tenocyte-like mRNA and protein profile, as well as active, yet controlled, collagen deposition.

## 4. Elastomeric Scaffolds and Biomimetic Materials

It is now well appreciated that seeded grafts vastly improve outcomes over unseeded grafts [57–59]. When Langer and Hubbell began using biomimetic self-assembling scaffolds, they determined that a successful graft must display several properties: (a) the scaffold should support cell adherence, (b) local growth factors should accumulate and be released when appropriate, and (c) the scaffold should be resistant to matrix proteases [63–66]. The success of many scaffolds has lent credence to these core concepts and should thus be an integral aspect of scaffold design. 

The inception of soft tissue regeneration efforts began with using small intestinal submucosa (SIS) as substitute for graft material harvested from the patient. SIS is a xenogenic membrane harvested from porcine jejunum [67]. Mechanical removal of the muscularis and mucosa yields a thin, translucent submucosa. Decellularization produces a dense, native collagen matrix that is readily usable for a multitude of tissue engineering fields. Since the recent Food and Drug Administration approval of this material, SIS has been used for rotator cuff reconstructions [68]. The collagen matrix in SIS is immediately ready for graft purposes, and the extracellular proteins (elastin, laminins, fibronectins, and proteoglycans) confer an additional layer of stability to the product. By removing cellular contents with peracetic acid, cytokines and growth factors of the digestive organ are removed to allow better acceptance in other sites like synovium. This strategy, however, violates current trends of using autologous sources of tissue. Harvesting SIS can elicit an immunologic reaction and diminish its applicability for patients since an amplified inflammatory response can lead to tissue damage and poor wound healing. SIS has been shown to undergo contracture *in vivo*, and the high batch-to-batch variability limits the potential in therapeutics. 

Recently, collagen matrices cultured with MSCs have appeared on the horizon for tendon repair [69–71]. The promising technique of isoelectric focusing aligns collagen fibers to the parameters of the target tissue, adjusting to the density, alignment, and strength of dense connective tissue. These electrochemically aligned collagen (ELAC) matrices support a higher proliferation rate of MSCs compared to randomly oriented collagen. The mere orientation of ELAC upregulates scleraxis and tenomodulin in MSCs, supporting the shift towards tenogenic differentiation of MSCs when presented with an aligned and dense collagen substrate. ELAC scaffolds, however, only fulfill the mechanical prerequisites of scaffold design. While collagen orientation and MSC adherence is important, ELAC does not support the incorporation of growth factors and cytokines needed in healing. 

### 4.1. Poly(1,8 octanediol-co-citrate) Scaffolds for Tendon Regeneration

Currently, the versatility of synthetic polymers shows great promise in tissue engineering. A novel material, first established in therapeutics by Ameer et al. [72], and subsequently by Sharma et al. [73], is presented here for consideration in tendon regeneration. Poly(1,8 octanediol-co-citrate) scaffold (POC) is a highly reproducible elastomeric material [72, 73] capable of being used as a synthetic scaffold to support cell growth. By means of comparison, a similar material called poly(lactide-co-glycolic acid) (PLGA) has been previously utilized to deliver stem cells for tendon regeneration efforts [74]. PLGA is capable of achieving an elastic modulus comparable to tendons (750 MPa), with a degradation pattern lasting seven weeks *in vitro*. 

The selection of POC over other compounds such as PLGA or poly-glycolic acid (PGA), however, stems beyond their composition. Both PLGA and POC are porous enough to support cell growth, and both degrade to similar nontoxic byproducts. Yet, POC is thinner allowing greater oxygen and fluid exchange (hence a greater delivery of nutrients to developing tissue) across opposing faces, which benefit cell proliferation in the long run. POC scaffolds can also be customized to a higher degree than PLGA, allowing the dynamics of elasticity to better reflect native ligament and tendon dynamics. Despite exhibiting equal support of robust cell growth, the three-dimensional PLGA structure does not suture well onto living tissue.

POC scaffolds provide adhesion substrates for anchoring cells and delivering growth factors through controlled release upon scaffold degradation [75]. POC scaffolds are biodegradable and resorbable—they degrade by non-enzymatic hydrolysis into CO_2_ and H_2_O. These polymers form highly adaptable and labile scaffolds due to their ester bonding scheme and are rapidly reproducible as well. While POC scaffolds lack any biological property, the polymerization conditions can be optimized so that POC scaffolds mimic the tensile strength and Young's modulus of collagen-based elastic tissue. Briefly, when equimolar amounts of citric acid and 1,8 octanediol are combined, melted, and cooled to make a prepolymer, polymerization dynamics can be thereafter adjusted based on temperature and time parameters [76]. High temperatures with short polymerization times yield denser thin films, while low temperatures and long polymerization times produce lower cross-linked films. These properties make POC a superior scaffold to non degradable constructs for two reasons. First, a second surgery to remove the device is not necessary, and non degradable scaffolds tend to not reproduce the mechanical behavior of the target tissue. Tables 2(a) and 2(b) exhibit the scaffolds presently available for therapeutic or experimental use [69, 73, 77–86].

The physical properties of POC can be exquisitely tailored for a variety of tissue morphologies and functions. For example, a thin layer of POC would have no difficulty serving as a graft for both round (Achilles) and flat (rotator cuff) tendons. Moreover, POC scaffolds would graft equally well onto unsheathed and sheathed tendons, without hindering smooth gliding. For tendons that angle around bony prominences, POC scaffolds would be flexible enough to maintain this conformation and remain intact throughout motion. The Young's modulus, measured according to Hooke's law under stress/strain conditions, is readily available for most tendon and ligamentous structures (Table 3). Hence, crafting POC scaffolds according to these elasticity and stiffness values allow for a consistent construction for specific anatomical targets. Additional parameters, such as Poisson's ratio, hysteresis, and creep, would further characterize the target tissue and allow for an even better matched scaffold design.

While the majority of tendon and ligament ruptures occur in the body of tendon, a proportion do occur at the fibrocartilage interface as well. In this region, collagen fibers blend into the bony attachment as perforating fibers (Sharpey fibers) that become continuous with the periosteum. During slowly increasing loading rates on the ligament, the insertion point of fibers becomes the weakest point. The importance of this junction has recently become a target for therapeutics as well, since weakness at this interface can jeopardize even the best efforts of reconstruction. A detailed discussion by Mikos et al. emphasizes that regeneration of the interface is a “prerequisite for achieving biological fixation of soft tissue grafts” [87]. In one study, MSCs were shown to accelerate the remodeling of the tendo osseous interface when implanted into bone tunnels [88], implying that MSCs have a potential in this region as well. 

Instead of attaching tendinous grafts to bone via screws, the optimal approach is reconstruction using the collaboration of synthetic materials with MSCs. Paradoxically, the very complexity of the fibrocartilage interface makes it a perfect candidate for POC utilization. Mimicking the strategy of Lu and colleagues [89], a scaffold with three distinct regions would allow formation of collagenous tendon along one edge, osseous material along the other, and a middle zone representing the transition from tendon to bone. Given the capacity of MSCs to differentiate into osteogenic and tenogenic lineages, a single cell population seeded onto the scaffold could regenerate the complex fibrocartilage interface. Additionally, POC scaffolds could be crafted according the target tendon interface, relying on Wolff's Law to govern the dynamics and load of the tendon aimed for reconstruction.

## 5. Growth Factors and Cytokines for Angiogenesis

While mature tendons are poorly vascularized and sustained by synovial fluid [90], developing tendons are highly vascular. The rich capillary network associated with tenogenesis arises mainly from the muscle-tendon junction, the osteotendinous junction, or from the surrounding connective tissue [91]. As described, tendons may revert to an embryonic state of intrinsic repair in order to lay down collagen after injury. From a vascular standpoint, it is not unexpected then that acutely injured tendons sprout capillary buds at the site of laceration [90]. Indeed, clinicians are careful to preserve the richly vascular connective tissue around the injury in order to surround the graft. Avoiding necrosis and supplementing the graft so that synovial fluid is not the only source of nutrition is key to graft survival [90, 92].

Vasculogenesis at the site of injury is dependent on VEGF [93]. Through tyrosine kinase receptors, VEGF guides hemangioblasts to differentiate into endothelial progenitors, which form new vessels to supply the site of injury. When angiogenic responses are induced by wounding, endothelial progenitors are rapidly mobilized. Aided by FGF-1, 2, TGF-*β*, PDGF, and TNF*α* [94, 95] from tenocytes and surrounding connective tissue, these cytokines and growth factors support tendon grafts and promote tissue remodeling. Results from embryologic studies reveal that TGF-*β* increases the transcription factor scleraxis [96]. Scleraxis is key to tendon maturation, and even in adenoviral-mediated transduction of MSCs with scleraxis gene, the tissue demonstrated improved stiffness, increased stress-to-failure levels, and a greater deposition of fibrocartilage [60]. A systematic review of pertinent growth factors adds cartilage-derived morphogenetic protein (CDMP), IGF, VEGF, IL-10, and FGF to the repertoire of factors necessary in tendon regeneration [97]. These factors are summarized in Table 4 [98–107].

Enhancing the reparative effects of a vascular network around the site of injury can be achieved through direct, localized delivery of proangiogenic factors. Efforts to transfect these factors into cells, or deliver factors through liposomal-mediated gene transfer, have been improvements to direct infusion of recombinant growth factors [108]. However, this approach may not be appropriate for the tendon microenvironment, since delivery schemes would not reach avascular regions of the tendon. Moreover, naked DNA transfection relies on cellular transcription factors to produce the angiogenic factors, placing great responsibility upon already damaged tissue to support itself.

The inherent properties of POC offer an alternative means of growth factor delivery. During the polymerization of POC, small kDa-sized growth factors may be locked within the scaffold and released upon surface erosion. In a study by Sharma et al. [75], elastomeric POC scaffolds were modified with heparan sulfate and loaded with VEGF, FGF2, and IGF-1 prior to rat implantation. These constructs released the pro-angiogenic growth factors through a systematic and controlled degradation and produced an increased vascular growth *in vivo* as compared to controls. Heparan sulfate, a highly sulfated glycosaminoglycan, serves to protect bound growth factors and extend their half-life. Such a construct would also permit the delivery of factors to modulate degradative enzymes in the healing tissue [16]. 

Alternatively, POC may be seeded with primitive stem or progenitor cells alongside MSCs. Sourced from the same origin as MSCs, this prevents unnecessary intrusion into patients, as a single bone marrow aspiration can yield ample cells of each population. Discovery and use of CD34+ hematopoietic stem cells (HSCs), expressing von Willebrand Factor (vWF), vascular endothelial-cadherin (VE-cadherin), and Flk-1, proved to increase neovascularization and reduce fibrosis when injected to the site of injury [109, 110]. These markers, in addition to CD133, CD34 and AC133 [111], allow for ease of isolation through flow cytometry. Given that endothelial progenitors are compatible with synthetic support matrices [112], blood vessel elongation and branching will benefit from the porosity of POC scaffolds as the cell-scaffold construct matures. Meticulous studies would have to be conducted to track the termination of neovascularization, since mature tendons are avascular. It is presumed that an overgrowth of vessels would hinder the stability and function of the graft. At present, the use of stem cells in orthopedic scaffolds is limited to animal studies only [16].

## 6. Present Barriers and Future Directions

The combinatorial effect of MSCs, POC scaffolds, and growth factors creates a strong approach to treating common tendon injuries. While Gulotta et al. [35] refutes the therapeutic effects of MSCs in tendon healing, our approach may suggest otherwise. Findings that state MSCs did not improve structure, composition, or strength of the tendon [35] can be attributed to the lack of growth factor use in the construct. Additionally, the delivery of cells through a fibrin matrix may not be a suitable substrate for MSC differentiation under *in vivo* conditions. 

Initiating a tendon regeneration process requires the identification and isolation of appropriate cell types that are able to proliferate and sustain growth over the healing process while maintaining physiological integrity of the graft. The function potential of MSCs and CD34+ HSCs is directly dependent on proper synthetic scaffold design. In consonance with POC scaffolds, we exploit the vast potential MSCs to lay down collagen and hematopoietic precursors to weave vessels through the tapestry of the scaffold. Reinforcements after surgery with therapy and NSAIDs have been shown to increase insoluble and total collagen, translating to increases in tensile strength and restoration [113]. 

Since POC can be customized to various densities, the application of such a versatile scaffold expands beyond orthopedics. Here, formation of dense regular connective tissue was discussed, but POC may farewell in regenerating dense irregular and loose areolar connective tissue as well. For example, POC scaffolds could contribute to three conditions that utilize tissue engineering: (a) dermal-epidermal reconstitution for burn victims with MSCs to supply fibroblasts and adipose tissue, (b) urinary bladder wall regeneration in neurogenic conditions with MSCs supplying contractile smooth muscle cells, and (c) cartilage formation for osteoarthritis and meniscal tears. In every case, the functional trio of MSCs, POC, and growth factors may one day supplement current surgical tactics.

## Figures and Tables

**Table 1:** Immunohistochemical comparison of MSCs and TDSCs.

Cellular marker	Bone-marrow-derived mesenchymal stem cells [47, 114–116]	Tendon-derived stem cells [117, 118]
CD18	−	−
CD31	−	−
CD34	−	−
CD40	−	−
CD44	+	+
CD45	−	−
CD90	+	+
CD90.2	−	+
CD105	+	+
CD106	+	−
CD117	−	−
CD144	+	−
CD146	+	+
Sca-1	−	+
Oct-4	+	+
SSEA-4	+	+
Stro-1	−	+
Nucleostemin	−	+
Flk-1	+	−
Tenomodulin	+	++
Scleraxis	+	++
Cartilage oligomeric protein (Comp)	+	++
Tenascin	+	++
Sox-9	+	+
Runx2	+	+
COL1	+	++
COL2	−	−
*α*-smooth muscle actin	++	+
Fibronectin	+	+

**Table:** (a)

Scaffold	Composition	Status	Support for growth factor release?
*Matrix xenografts*			
Restore	Porcine SIS	FDA approved	Yes (TGF-*β*, VEGF, FGF-2)
CuffPatch	Porcine SIS	FDA approved	Yes (EGF, TGF-*β*, FGF)
TissueMend	Bovine dermal extracellular matrix	FDA approved	No
Zimmer collagen repair patch	Porcine acellular dermal matrix	FDA approved	No
Permacol	Porcine acellular dermal matrix	FDA approved	No
Conexa	Porcine acellular dermal matrix	Experimental	No

*Matrix allografts*			
GraftJacket	Human acellular dermal matrix	FDA approved	Yes

**Table:** (b)

Scaffold	Status	Support for growth factor release?
*Non degradable synthetic polymers*		
Polyethylene terephthalate (Stryker-Dacron)	FDA approved	No
Polypropylene (Kennedy Ligament Augmentation Device)	FDA approved	Yes
Poly(tetrafluoro ethylene) (GoreTex)	FDA approved	Yes

*Biodegradable Synthetic Polymers*		
Polylactic acid	FDA approved	Yes
Polyglycolic acid	FDA approved	Yes
Poly(lactide-co-glycolic acid)	FDA approved	Yes
Polydioxanone	FDA approved	No
Polycaprolactone	FDA approved	Yes
Hydrothane/PET	FDA approved	
x-Repair Device (poly-L-lactide)	FDA approved	Yes
Hyaluronan-based non woven mesh (HYAFF11)	Experimental	Yes
Electrochemically aligned collagen matrices (ELAC)	Experimental	No
Poly(1,8-octanediol-co-citrate)	Experimental	Yes

*Nanofiber matrices*		
Peptide Amphiphiles	Experimental	Yes

**Table 3:** Young's Modulus of Human Tendons and Ligaments. Young's Modulus = stress/strain = (*F*/*A*)/(Δ*L*/*L*_*o*_).

Tendon or ligament	Young's modulus (MPa)	Reference
Gracilis	612.8	Butler et al. [119]
Semitendinous	362.2	Butler et al. [119]
Patellar	1090	Hansen et al. [120]
Lateral collateral ligament	350–400	Butler et al. [121]
Posterior cruciate ligament	300–400	Butler et al. [121]
Anterior cruciate ligament	300–350	Butler et al. [121]
Tibialis Anterior	1200	Maganaris and Paul [122]
Infraspinatus	527	Halder et al. [123]
Teres minor	14	Halder et al. [123]
Gastrocnemius tendon	1160	Maganaris et al. [123, 124]

**Table 4:** Summary of growth factors necessary in tendon regeneration.

Growth factor	Size (kDa)	Function
TGF-*β*	25	Promotes angiogenesis and collagen production
EGF	6.4	Mitogenic to fibroblasts and promotes collagenase activity to remodel the extracellular matrix
PDGF-*β*	12.3	Mitogenic to fibroblasts, chemoattractant to macrophages, and assists angiogenesis
bFGF	22–24	Released from extracellular matrix to promote angiogenesis and granulation
VEGF	38.2	Vasculogenesis and angiogenesis during tissue hypoxia
Hepatocyte growth factor (HGF)	*α* chain: 69 *β* chain: 32–34	Expressed in wound fibroblasts to regulate growth, motility, and morphogenesis
BMP-12,13,14	30–38	Promotes tendon-derived stem cell differentiation into tenocytes
Early growth response factor-1 (EGR-1)	75	Transcription factor that upregulates collagen and accelerates wound closure
